# A web-based survey on various symptoms of computer vision syndrome and the genetic understanding based on a multi-trait genome-wide association study

**DOI:** 10.1038/s41598-021-88827-y

**Published:** 2021-05-03

**Authors:** Keito Yoshimura, Yuji Morita, Kenji Konomi, Sachiko Ishida, Daisuke Fujiwara, Keisuke Kobayashi, Masami Tanaka

**Affiliations:** 1 DeNA Life Science, Inc., Tokyo, Japan; 2 Kirin Central Research Institute, Kirin Holdings Company, Limited, Yokohama, Japan; 3 Clinical and Translational Research Center, Keio University Hospital, Tokyo, Japan; 4 Health Science Department, Kirin Holdings Company, Limited, Tokyo, Japan

**Keywords:** Eye manifestations, Statistical methods, Disease genetics, Genetic variation

## Abstract

A variety of eye-related symptoms due to the overuse of digital devices is collectively referred to as computer vision syndrome (CVS). In this study, a web-based survey about mind and body functions, including eye strain, was conducted on 1998 Japanese volunteers. To investigate the biological mechanisms behind CVS, a multi-trait genome-wide association study (GWAS), a multivariate analysis on individual-level multivariate data, was performed based on the structural equation modeling methodology assuming a causal pathway for a genetic variant to influence each symptom via a single common latent variable. Twelve loci containing lead variants with a suggestive level of significance were identified. Two loci showed relatively strong signals and were associated with TRABD2B relative to the Wnt signaling pathway and SDK1 having neuronal adhesion and immune functions, respectively. By utilizing publicly available eQTL data, colocalization between GWAS and eQTL signals for four loci was detected, and a locus on 2p25.3 showed a strong colocalization (PPH_4_ > 0.9) on retinal MYT1L, known to play an important role in neuronal differentiation. This study suggested that the use of multivariate questionnaire data and multi-trait GWAS can lead to biologically reasonable findings and enhance our genetic understanding of complex relationships among symptoms related to CVS.

## Introduction

With the growth of digital devices, there are many complaints nowadays about a variety of eye-related symptoms such as eye strain, blurred vision, and double vision, and it is collectively referred to as digital eye strain or computer vision syndrome (CVS). In addition, it is also known to be associated with non-ocular symptoms such as headaches, and shoulder and neck pain. The American Optometric Association has defined CVS as a combination of eye and vision problems linked to the use of visual display terminals (VDTs)^1^. The diagnosis is carried out based on a comprehensive examination including visual acuity measurements, testing for refractive error and focusing movement in addition to medical inquiries about symptoms the patient has experienced. Although CVS is associated with a variety of symptoms, Sheedy et al.^2^ stated that the underlying problem can be identified by the location of the symptoms and categorized into the two groups of internal and external symptoms. According to the general review by Blehm et al.^3^, there are four types of symptoms (asthenopic, ocular surface-related, visual, and extra-ocular) and they can be subdivided into three main potential pathophysiological causes (ocular surface mechanisms, accommodative mechanisms, and extraocular mechanisms). Numerous studies have been conducted so far to address health and safety issues for VDT users, but the efficacy of proposed treatments for minimizing CVS-related symptoms have not been adequately demonstrated yet^3,4^. A better understanding of the underlying physiology behind CVS is important to enable more accurate diagnosis and treatment.

The genome-wide association study (GWAS)^5^ has become a common genetic approach to identify potential susceptibility genes for diseases. To date, a number of GWAS have been successful in identifying the genetic pathways associated with ocular diseases such as myopia, age-related macular degeneration and glaucoma^6,7^. Although GWAS has brought forth an adequate amount of knowledge on the genetic etiology of ophthalmic traits, through relatively easy to recruit severe cases, more common diseases or syndromes will require an increase in sample size and well-designed analytical methods. No GWAS therefore has been reported for CVS, and we need an elaborate method to integrate multiple symptoms related to CVS, which cannot be directory measured, into a model in order to precisely evaluate genetic effects using GWAS.

With the establishment of cohorts with more detailed phenotypic information, multi-trait GWAS, which is a multivariate analysis on the same subjects, has also become possible. Multivariate methods are generally considered more powerful than univariate methods unless only one trait is associated with a genetic marker or all traits are very highly correlated^8^. Many methods have been proposed and applied so far, including dimensionality reduction using principal component analysis (PCA), multivariate mixed models as an extension of regression analysis, Bayesian polynomial regression and so on^8,9^. In recent years, methodologies using structural equation modeling (SEM) have been applied for multi-trait GWAS^10,11^. SEM is a multivariate statistical method in which the interactions between variables are described as simultaneous equations and their parameters are inferred^12^. Unlike other methods, the SEM-based method can incorporate latent variables, which are hypothetical quantities that have not been measured and can be inferred from other measured variables, into the model. A SEM-based multi-trait GWAS is attained by assuming a model structure in which a genomic variant is an exogenous predictor that influences observed variables via the latent factor, and then repeating the statistical test genome-wide for each variant. SEM has enabled investigations of complex traits such as psychology and psychiatry, in which their unmeasurable phenotypes are indexed by multiple symptoms or behaviors. Since this feature is similar to CVS, we applied a SEM-based multi-trait GWAS to CVS, which we expected increased power to identify genetic associations.

In this study, a web-based survey about CVS was conducted on 1998 Japanese volunteers. To investigate the biological mechanisms behind CVS, we performed the SEM-based multi-trait GWAS for the CVS-related multiple symptoms for the first time to the best of our knowledge under the assumption of the existence of a potential phenotype behind the CVS-related symptoms.

## Methods

### Study population and data collection

Among customers of a direct-to-consumer (DTC) genetic testing service (MYCODE), 1998 Japanese volunteers (50.5% male, mean age 49.3 years) were recruited in August 2019 (Table 1). Customers with prior consent in writing for research recruitment were invited to participate in this study via the company website and email. Participation was voluntary and based on informed consent. The web-based questionnaire survey was conducted with the participants. A total of 14 questionnaire items covering the four categories defined by Blehm et al.^3^ were designed (Table 2). Each item was measured on a scale of one to seven in each of the two conditions: “When you feel like you have used your eyes” and “When you feel like you have not used your eyes very much”. These two conditions were set to simulate the answers before and after the VDT task, and the difference (delta) of these two conditional answers was used in the following analysis. In addition to that, self-reported medical history was obtained on the presence or absence of dry eye syndrome, presbyopia and other common eye diseases. This study received approval from the institutional review board of DeNA Life Science, Inc. and was implemented in accordance with the Declaration of Helsinki.

**Table 1:** Characteristics of participants in this study.

	Age group (years)						Overall n = 1998
	20–29 n = 51	30–39 n = 287	40–49 n = 661	50–59 n = 702	60–69 n = 249	≧70 n = 48	
Male, n (%)	24 (47.1)	145 (50.5)	290 (43.9)	364 (51.9)	151 (60.6)	35 (72.9)	1009 (50.5)
**Eye strain questionnaire, mean ± SD**^**a**^							
Self-aware before VDT	2.08 ± 1.18	1.97 ± 1.22	2.00 ± 1.24	2.10 ± 1.30	2.17 ± 1.33	1.92 ± 1.40	2.05 ± 1.27
Self-aware after VDT	5.35 ± 1.48	4.86 ± 1.60	4.83 ± 1.76	4.72 ± 1.70	4.21 ± 1.62	3.65 ± 1.86	4.70 ± 1.71
**Dry eye questionnaires**^**b**^							
DEQS, mean ± SD	29.7 ± 22.0	23.2 ± 17.6	25.3 ± 19.9	26.8 ± 20.1	21.5 ± 17.3	21.6 ± 19.5	25.1 ± 19.5
DEQS (> 15), n (%)	33 (64.7)	165 (57.5)	386 (58.4)	455 (64.8)	131 (52.6)	24 (50.0)	1194 (59.8)
**Presbyopia questionnaires, n (%)**							
self-aware	0 (0.0)	13 (4.5)	363 (54.9)	607 (86.5)	207 (83.1)	37 (77.1)	1227 (61.4)
Glasses use	0 (0.0)	2 (0.7)	72 (10.9)	293 (41.7)	122 (49.0)	22 (45.8)	511 (25.6)
**Other questionnaires, n (%)**							
VDT hours per a day (≥7 h)	27 (52.9)	136 (47.4)	243 (36.8)	201 (28.6)	35 (14.1)	2 (4.2)	644 (32.2)
Daily glasses and/or contact lens use	35 (68.6)	196 (68.3)	439 (66.4)	504 (71.8)	200 (80.3)	42 (87.5)	1416 (70.9)
Daily eye drop use	18 (35.3)	99 (34.5)	262 (39.6)	284 (40.5)	124 (49.8)	23 (47.9)	810 (40.5)
**Self-reported concomitant diseases, n (%)**							
Dry eye syndrome	8 (15.7)	53 (18.5)	122 (18.5)	133 (18.9)	50 (20.1)	12 (25.0)	378 (18.9)
Severe myopia	2 (3.9)	27 (9.4)	53 (8.0)	61 (8.7)	15 (6.0)	8 (16.7)	166 (8.3)
Cataract	0 (0.0)	2 (0.7)	10 (1.5)	44 (6.3)	40 (16.1)	19 (39.6)	115 (5.8)
Glaucoma	0 (0.0)	6 (2.1)	30 (4.5)	44 (6.3)	23 (9.2)	8 (16.7)	111 (5.6)

This is a relatively older population, but it can be seen to cover all age groups to some extent. ^a^It is simulated to responses before and after the VDT task by setting conditions of eye use. ^b^Based on the dry eye-related quality-of-life score QOL (DEQS) questionnaires^42^, participants who were likely positive for dry eye syndrome were screened at a cutoff of > 15 DEQS^43^.

*SD* standard deviation.

**Table 2:** List of CVS-related questionnaires and statistics on responses.

	Symptom category	Questionnaire item	Symptomatic responses (> 4 of 1–7), n (%)	
			Before VDT	After VDT
q1	Asthenopic	Eye strain	113 (5.7)	1153 (57.7)
q2		Sore eye	63 (3.2)	474 (23.7)
q3		Eyelid pain and heavy feeling	58 (2.9)	373 (18.7)
q4	Ocular surface-related	Dry eyes	211 (10.6)	741 (37.1)
q5		Watery eyes	75 (3.8)	275 (13.8)
q6		Irritated eyes	97 (4.9)	425 (21.3)
q7		Red eyes	83 (4.2)	434 (21.7)
q8	Visual	Blurred vision	160 (8.0)	898 (44.9)
q9		Double vision	51 (2.6)	346 (17.3)
q10	Extra-ocular	Tired body	134 (6.7)	573 (28.7)
q11		Back and shoulder pain	324 (16.2)	1003 (50.2)
q12		Headache	67 (3.4)	494 (24.7)
q13		Heavy head	46 (2.3)	438 (21.9)
q14		Annoyance	43 (2.2)	355 (17.8)

“Eye strain”, “blurred vision”, and “back and shoulder pain” have also been reported as common symptoms in digital device users from other studies^44,45^. In contrast, the prevalence of “headache” in this study were relatively lower, but this corresponds to another study on Japanese population^46^.

### Genotyping and quality control

Participants collected their own saliva samples using the kit sent to them when they used the service before participation in this study, and DNA extraction and genotyping were performed at the laboratory of DeNA Life Science, Inc. Infinium OmniExpress-24 or Human OmniExpress-24 BeadChip (Illumina, Inc.), both of which additionally contained approximately 30,000 custom probes, was used. The stringent quality control (QC) procedures of genotyped results were applied with call rate ≥ 95%, minor allele frequency (MAF) ≥ 0.01, identity by descent < 0.1875, and Hardy–Weinberg equilibrium (p value > 1.0e−6). The gender on the declaration was confirmed to match the genotyped one. All the above procedures were performed using PLINK (ver. 1.9)^13^. In order to stratify the population, PCA computed the first two principal components based on a sample set of 1000 Genomes Project phase 3 (1KGP; N = 2504) and then projected study samples onto the 2D subspace. Samples falling outside the Japanese cluster were excluded (Supplementary Fig. S1). Eventually, 1966 subjects (999 male) with a total of 540,916 single nucleotide polymorphisms (SNPs) on autosomes ended up passing the QC filter. GRCh37/hg19 (GCF_000001405.13) was the reference for the genome construction information.

### Statistical analysis

To implement the multi-trait GWAS based on the SEM methodology, gwsem package (ver. 0.1.17) in R, which was developed by Verhulst et al.^14^, was introduced. Age, sex, and the top 2 eigenvectors from PCA were added into the model as external covariates. The number of principal components to be incorporated was determined based on examination of the scree plot of their eigenvalues. After running the SEM-based multi-trait association analysis on all variants, p values were subsequently calculated, adjusting each test statistic by a genomic inflation factor. Genotype imputation was conducted on variants which were located within ± 500 kb of GWAS hits, and SEM-based GWAS was rerun on estimated genotypes. Eagle2 (ver. 2.4.1)^15^ was used for the phasing step referring to the East Asian population (EAS; N = 504) of 1KGP, and then imputation was performed using Minimac3 (ver. 2.0.1)^16^. The accuracy index of imputation performance, correlation between reference and predicted genotypes (R squared), was 0.942 ± 0.116 for common variants (MAF ≥ 0.05; see Supplementary Fig. S2). Imputed variants with R squared < 0.7 were removed for QC purpose. GWAS hits meeting a suggestive level of significance (p value < 1.0e−5) were considered lead variants, and the locus was defined as a region containing highly correlated variants (R squared > 0.6) with each lead variant in the 1KGP EAS population.

Colocalization between GWAS summary statistics and expressed quantitative trait locus (eQTL) signals was analyzed based on a Bayesian statistical methodology proposed by Giambartolomei et al.^17^ We utilized GSE115828 data (N = 523)^18^, the only publicly available retinal tissue eQTL data from the Gene Expression Omnibus (GEO) of the National Center for Biotechnology Information as of May 2020. The whole blood eQTL data in the Genotype-Tissue Expression (GTEx ver. 7; N = 369)^19^ was analyzed as well. In addition, two data sources for eQTL data (GSE53351^20^ and hum0099.v1^21^) were added, available from GEO and the National Bioscience Database Center and consisting of more than 100 healthy Japanese subjects. The former is whole blood data (N = 301) and the latter is eQTL data from whole blood cells and five immune cell populations (CD4^+^ T cells, CD8^+^ T cells, B cells, NK cells, and monocytes) of 105 subjects. Note that previous studies^19,22^ have demonstrated that a sample size of about 100 individuals is sufficient to detect major eQTL effects in various cell types collected in a single population. Posterior probability of H_4_ (PPH_4_) of colocalization was calculated for each ± 100 kb of the identified loci containing lead variants using the coloc package in R.

## Results

### Model specification via cause-and-effect graph

**Figure 1:**
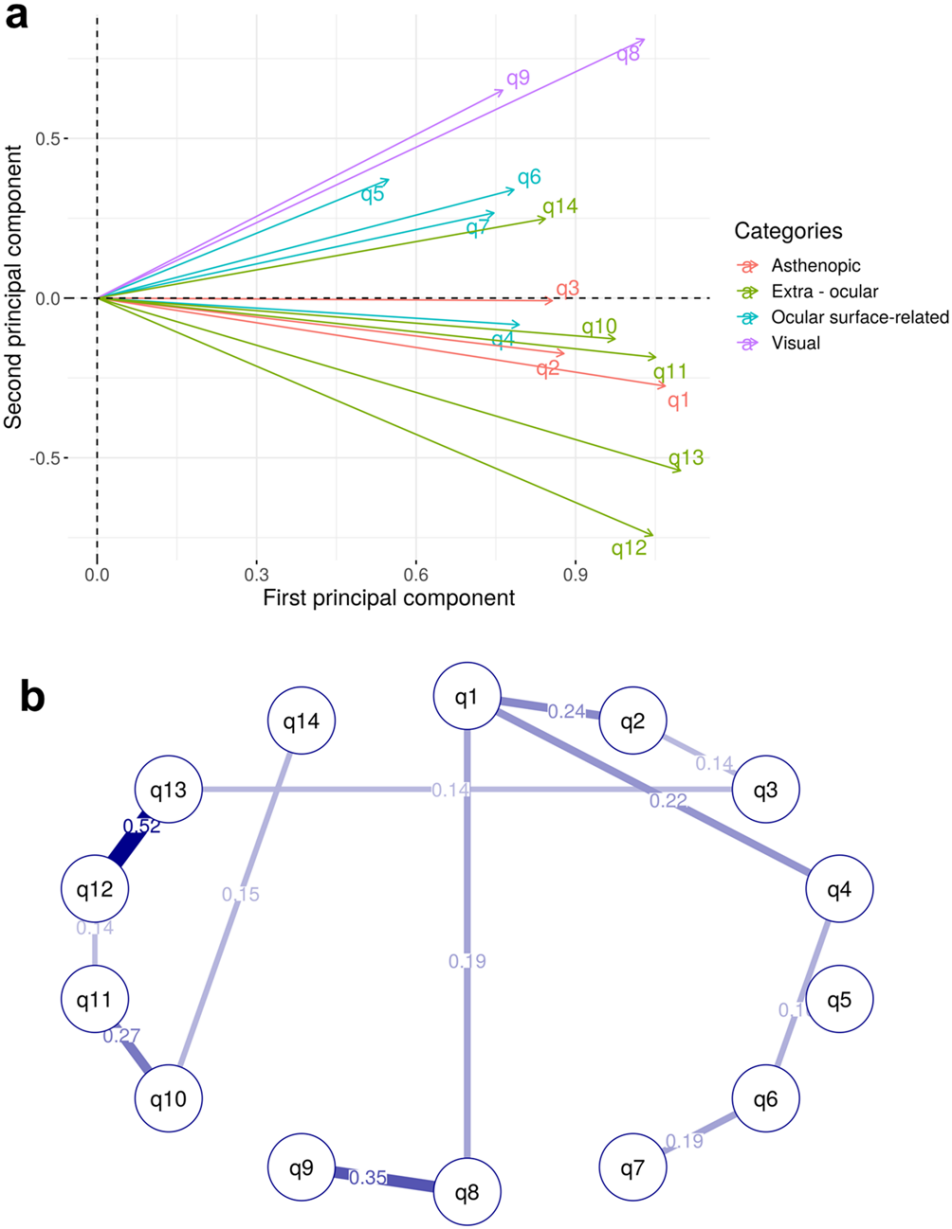
(**a**) PCA results for questionnaire items based on the partial correlation matrix. The length of arrows indicates contributions of the variables to the principal axes. The proportion of variance captured is given as a percentage for both the first and second principal components. (**b**) Partial correlation network estimated by covariance selection. A dense subnetwork of high partial correlated edges is called a clique, and the existence of a clique would suggest the presence of the same number of latent factors. No cliques with edges all having values greater than 0.2 were found in the network. For model optimization, gmm package (ver. 1.6-5) in R was used to minimize Akaike information criterion.

**Figure 2:**
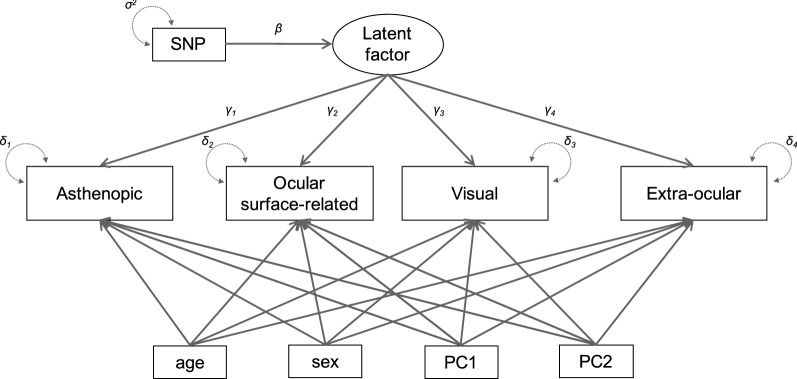
Node diagram of SEM for multi-trait GWAS. The observed variables (four symptom categories) are presented as rectangles and the latent variable as ellipses. Note that each symptom category was calculated by the average of component questionnaires. The association between the latent factor and the observed variables are estimated by the factor loadings (γ_k_). GWAS analysis infer the strength of the causal effect (β) from each SNP to the phenotypes via the latent variable. Age and gender, as well as the first two principal components of the PCA performed for population stratification, were added as covariates. The arrows describe the relationships between the variables. Residuals (δ_k_) and variances (σ) are drawn as double headed dashed arrows into an object.

It is assumed that the 14 questionnaire items are observed symptoms due to a potential syndrome, that is CVS, and multiple items will be observed in conjunction and correlated to some extent. The results of 1998 respondents were used to calculate pairwise partial correlation coefficients between items, and positive correlations among all items were verified (Supplementary Fig. S3). The PCA calculated from the eigenvalue vectors of the partial correlation matrix showed that all items were in the same direction on the first PC, and they appeared to form clusters corresponding to the four categories to which each item belonged on the second PC, though not completely (Fig. 1a). Next, covariance selection^23^, a graphical modeling technique, was applied to derive a partial correlation network consisting of only well-fitting edges between items by stepwise removing edges with low partial correlation coefficients (Fig. 1b). Although q5 "watery eyes" was a singleton that did not share edges with any of the items, it could be seen as forming one large network as a whole. Since the above suggests that each of the four categories captures its own characteristics among belonging items, the items were integrated (averaged) and the representative values of each category were used in the following analysis. In addition, we constructed a factor analysis model with one common factor for all categories (Fig. 2). Using OpenMx package (ver. 2.17.3) in R to calculate goodness of model fit, the Chi-squared statistic was 22.5 (degrees of freedom = 9; p value = 7.4e−3) and the root mean square error of approximation (RMSEA) was 0.028 (confidence interval 0.00–0.045). The closer the RMSEA is to zero, the better the fit to SEM, and a value below 0.05 is desirable^24^. This model was considered to be robust for SEM-based multi-trait GWAS.

### Multi-trait GWAS

**Figure 3:**
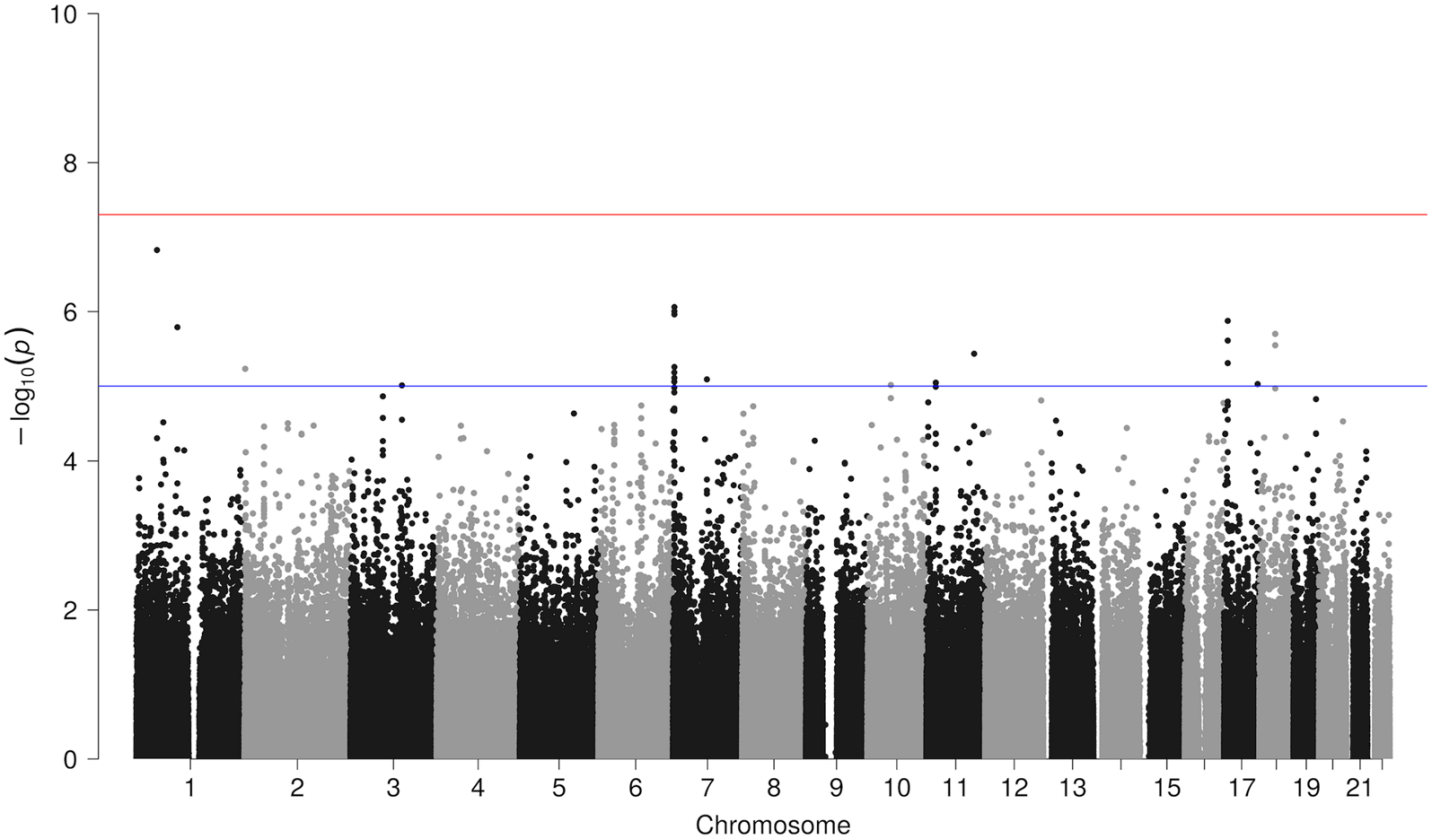
Manhattan plot of multi-trait GWAS results. The more significant the association, the higher the "skyscraper.” The red horizontal line represents the genome-wide significance threshold, while the blue line corresponds to the suggestive threshold.

SEM is a form of graphical modeling that uses graphs to assume causalities between observed and latent variables and estimates the strength of the causalities. We introduced a latent variable into the model, where the causal effect of each variant on the questionnaire items (observed variables) was transmitted via the latent variable (Fig. 2). Comprehensive association analysis on each variant was performed to infer the strength of association between the variant and latent factor, and the statistical significance was calculated as a p value. A quantile–quartile (Q–Q) plot was generated between the actual observed p values and the p values as theoretically expected, showing no significant population stratification (Supplementary Fig. S4). The output of GWAS can be represented by a diagram called a Manhattan plot, where each variant’s strength of association (− log10 of p value) is relative to the genomic position (Fig. 3). Although there were no hits with the genome-wide significance level (5.0e−8), 12 peaks with variants at the top that met the suggestive level of significance were detected (Table 3). For each of the peaks, regional association plots were generated using LocusZoom (ver. 1.4)^25^, and they all formed a single peak at each close location on the genome (Supplementary Fig. S5). When GWAS was performed again with the addition of other covariates (daily VDT hours, glasses and/or contact lens use, and eye drop use), there was no noticeable change in the relative trend, although the overall signal weakened slightly.

**Table 3:** List of identified loci via multi-trait GWAS.

Chr	GWAS Locus start	GWAS Locus end	GWAS Lead SNP	GWAS p value	MAF	Gene^a^	Functional consequence
1	48,233,438	48,233,438	rs12409657	1.5e−7	0.455	AL691459.1	upstream_gene_variant
						TRABD2B	intron_variant
1	95,074,455	95,074,455	rs1245489	1.6e−6	0.374	SLC44A3-AS1	downstream_gene_variant
2	1,625,705	1,627,501	rs9752743	5.9e−6	0.464	AC144450.1	intron_variant, non_coding_transcript_variant
3	118,409,090	118,423,392	rs9859270	9.8e−6	0.412	AC068633.1	intron_variant, non_coding_transcript_variant
7	3,319,615	3,715,120	rs9791502	8.7e−7	0.378	SDK1	intron_variant
7	78,236,116	78,262,175	rs38098	8.1e−6	0.497	MAGI2	intron_variant
10	54,053,817	54,064,531	rs7916906	9.7e−6	0.261	PRKG1	downstream_gene_variant
						PRKG1-AS1	downstream_gene_variant
11	21,774,416	21,901,738	rs4617585	9.0e−6	0.390	ANO5	intron_variant, non_coding_transcript_variant
11	109,931,409	109,937,812	rs12419121	3.7e−6	0.201	RDX	intron_variant, NMD_transcript_variant
17	8,611,269	8,637,070	rs2101938	1.3e−6	0.266	–	–
17	77,199,681	77,202,887	rs4789955	9.4e−6	0.416	RBFOX3	intron_variant
18	36,505,318	36,510,996	rs9945284	2.0e−6	0.446	–	–

^a^Possibly related genes predicted by the Ensembl Variant Effect Predictor^47^.

We performed single-trait GWAS on each of the four categories using gwsem, and several peaks detected by the multi-trait GWAS were identified as well (Supplementary Fig. S6). As an example, the peak upstream of the sidekick cell adhesion molecule 1 (SDK1) gene on 7p22.2 are seen in the "ocular surface-related" and "visual" categories, while the others also show a less significant association. Furthermore, since similarly consistent peaks can be found using PLINK, which is a gold standard tool for GWAS, there is little concern about bias due to the statistical processing method.

### GWAS and eQTL colocalization analysis

**Figure 4:**
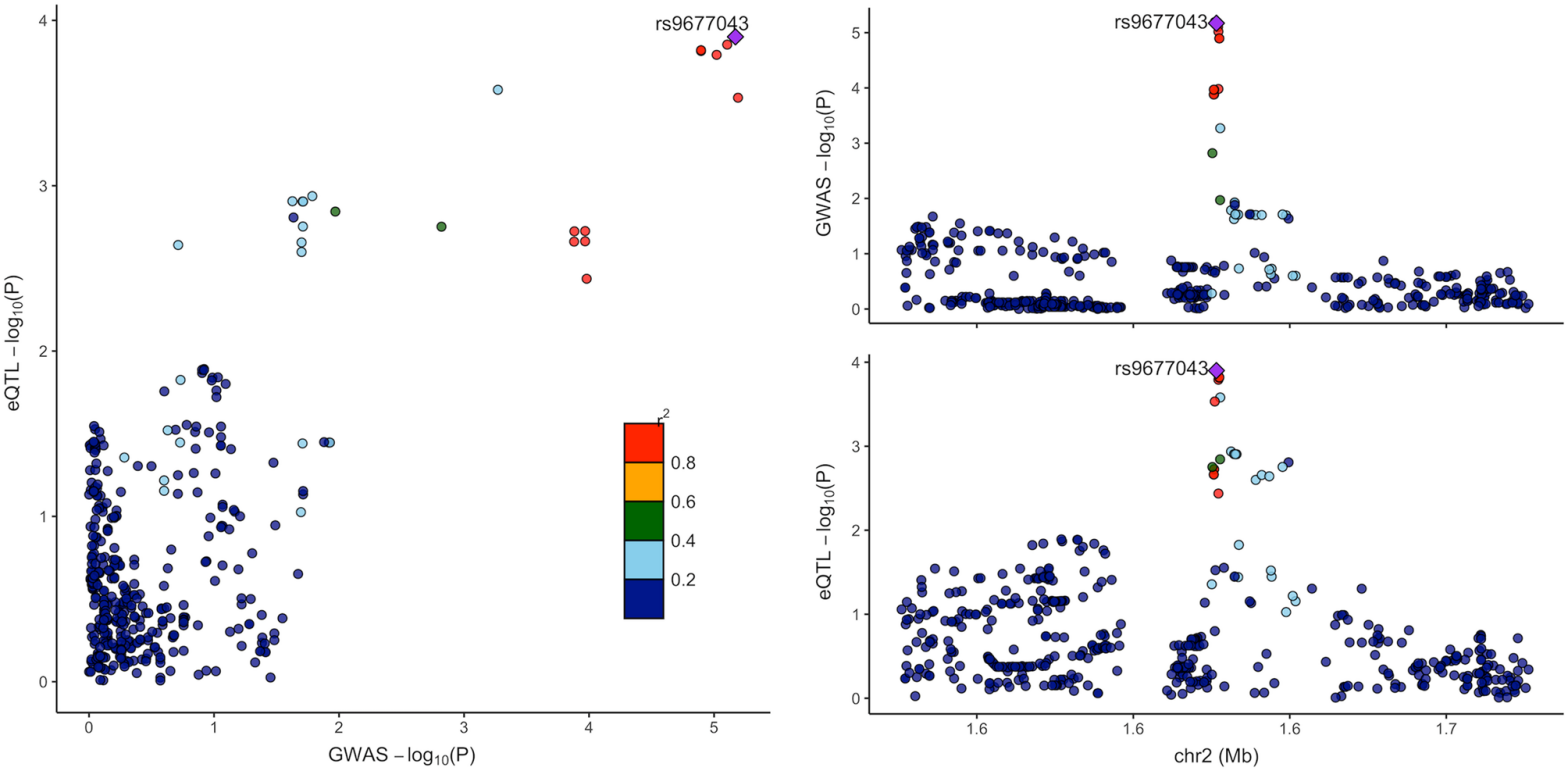
Regional plots of GWAS and eQTL signals on MYT1L gene in retina. Variants are colored based on their correlation with the labeled top variant, which has the smallest p value in the region.

None of the lead variants detected from multi-trait GWAS were located on a protein coding region. We examined the influence of detected variants on gene expression levels by utilizing publicly available eQTL data sources. In order to identify genes for which the expression level is affected by each variant, we focused on colocalization between the distribution of GWAS and eQTL signals. In other words, the p value of the GWAS result for each variant was compared with that of eQTL on a genome scale to see if they both show significant p values at the same position. Calculating a posterior probability (PPH_4_) when GWAS and eQTL are associated and share a single causal variant, combinations of locus, genes and expression tissue with > 0.5 of PPH_4_ were comprehensively explored. As a result, four combinations were detected (Table 4). When using a criteria > 0.75 of PPH_4_ strongly suggesting GWAS and eQTL signals are due to a common causal variant^26^, a strong colocalization (PPH_4_ = 0.906) between GWAS signals at the locus (1,625,705–1,627,501) ± 100 kb on chromosome 2 and eQTL signals on the myelin transcription factor 1 like (MYT1L) gene in retinal tissue was detected. As seen in the regional association plots generated by the locuscomparer package (ver. 1.0.0)^27^ in R, the p values distribution in GWAS and eQTL signals showed a similar shape and the high degree of correlation was visually confirmed as well (Fig. 4). This suggests that rs9677043, which has the strongest p value for both GWAS and eQTL, is a highly possible causal variant. Note that rs9677043 is located approximately 166 kb downstream of the MYT1L gene. We analyzed the association of rs9677043 with other self-reported diseases (presbyopia, dry eye syndrome, severe myopia, cataract, and glaucoma), but no significant associations were found.

**Table 4:** List of loci with a high colocalization identified via GWAS and eQTL colocalization analysis.

Chr	Locus Start	Locus End	Gene	Conditional probability of having colocalization in relevant tissue (PPH_4_ via coloc)								
				European		Japanese						
				GSE115828 (N = 523)	GTEx v7 (N = 369)	GSE53351 (N = 301)	hum0099.v1 (N = 105)					
				Retina	Whole blood	Whole blood	Whole blood	CD4^+^	CD8^+^	B	NK	Mono
1	48,233,438	48,233,438	FOXD2-AS1	0.048	0.037	–	–	**0.530**	0.011	0.000	0.000	–
1	95,074,455	95,074,455	ABCA4	**0.633**	–	0.036	–	–	–	–	0.002	–
2	1,625,705	1,627,501	MYT1L	**0.906**	–	0.020	0.001	0.000	0.000	–	–	–
11	21,774,416	21,901,738	ANO5	0.033	**0.716**	0.120	0.000	–	**0.516**	–	0.494	0.161

A hyphen stands for no feature in the eQTL data. The figures in bold are above 0.5 of PPH_4_.

*CD4*^*+*^ CD4^+^ T cells, *CD8*^*+*^ CD8^+^ T cells, *B* B cells, *NK* NK cells, *Mono* monocytes.

## Discussion

The MYT1L gene, the expression level of which showed a strong colocalization (PPH_4_ > 0.9) in retina, is a paralog of MYT1 and encodes a member of the zinc finger superfamily of transcription factors. It is mainly expressed in neural tissue and is known to play an important role in neuronal differentiation by specifically suppressing the expression of non-neuronal genes^28^. The eQTL data showed rs9677043, detected as a possible causal variant, has a positive effect on MYT1L expression levels in retinal tissue. The path coefficient β estimated by SEM was 0.132, showing that the mutation of rs9677043 worsens the observed symptoms. This suggests that the differentiation of neurons in retinal tissue has a potential to be associated with CVS.

Looking at other identified loci, Chr1:48,233,438–48,233,438 and Chr7:3,319,615–3,715,120 have relatively strong GWAS signals (p value < 1.0e−6), and they were associated with the TraB domain containing 2B (TRABD2B) and SDK1 genes according to the position on the genome, respectively. TRABD2B is a metalloproteinase having the function of a negative regulator of the Wnt signaling pathway, which has been reported to regulate the transdifferentiation of retinal nerves into the ciliary body^29^. On the other hand, SDK1 encodes a protein in the immunoglobulin superfamily taking a critical part of the immune response, and in addition, is thought to work in selective synapse formation between retinal neurons because of its ability to adhere specifically to another SDK1 on the surface of other cells^30^. All of these genes are associated with retinal nerves or a wide range of neural tissues. As chronic inflammation is reported to be one factor in neuronal dysfunction in the retina^31,32^, immune response also might provide clues to investigate the physiology of CVS.

We will describe the limitations of this study from the following four of viewpoints: (1) self-reported questionnaires via a web browser, (2) sufficiency of the level of statistical significance, (3) impact of racial differences in eQTL analysis, (4) need for validation study using different sample groups.

There is no doubt that web-based questionnaires are less clinically reliable than diagnosis by a doctor. Furthermore, it has been pointed out that there are some problems, such as the bias caused by the web-mediated process, compared to traditional laboratory questionnaire interviews^33^. On the other hand, one of the DTC genetic testing services, 23andMe in the US, has uncovered numerous biological findings utilizing self-reported medical history and web-based questionnaires from a large group of customers^34^. This is a new research approach that is driven by mass data and this study is a continuation of that trend.

The ordinary GWAS has established a very strict criteria—a genome-wide significance level. This study instead used the suggestive significance level to give a biological interpretation. The main reasons for the low statistical power may be the subjective way in which the trait was assessed, the wide spectrum of traits, and the small number of samples. Considering the original purpose of GWAS, we should find its meaning and value in the discovery of unknown important molecular mechanisms or new markers for diagnosis. In fact, other studies have led to an extended list of associated variants, which can provide a resource for functional studies, by using more exploratory criteria^35^. This requires sufficiently narrowing the window for the possibility of false positives, and this study used colocalization with eQTL to give additional validity to biological certainty.

It has been noted that the expression levels of many genes are variable between human populations, and it is mainly explained by differences in genotype frequencies^20^. We used HaploReg (ver. 4.1)^36^ to check rs9677043 detected from the European retinal eQTL data, and the MAF in the European and East Asian populations were 0.53 and 0.47, respectively. Although there is no large difference compared to this study (0.464), it is desirable to obtain retinal tissue eQTL data from the Japanese population, considering the fact that transcriptional regulation at the gene level cannot be explained by a single variant alone.

GWAS can be positioned as a “forward genetic” experiment to identify the genetic basis of a trait and provide an opportunity for exploratory hypothesis generation. In order to strengthen the hypothesis, it is ideal to conduct external validation on different sample groups, and this study is no exception. On the other hand, the common study design of examining the functional role of genetic variation is to knock out a specific gene in a model organism. Such an experiment based on “reverse genetics” is necessary for hypothesis verification. In recent years, national biobanks and DTC genetic testing services provide a large-scale cohort linked to genetic and rich phenotypic information, mainly obtained from electronic medical records. An approach of extending GWAS to the entire set of phenotypic entities is called the phenome-wide association study (PheWAS)^37^, and it allows “reverse genetic” experiments to be virtually conducted on human subjects^38,39^. The proof-of-principle for the hypotheses generated in this study is expected by making use of cohorts in the future. Besides, the Mendelian randomization approach is an extension of PheWAS to test for causal relationships between phenotypes, and the SEM methodology has been used in many cases^40,41^.

Last of all, we will discuss the benefits of the use of SEM for multi-trait GWAS. The path coefficients estimated by SEM reflect the strength of each causal relationship and quantify the influence of a variant on the target phenotype via intermediate variables (latent factors) and predefined causal paths. For instance, the positive path coefficient from an intermediate variable to an observed variable suggests that an increase in the intermediate variable directly leads to an increase in the observed variable. In the case of this study, the coefficients (γ) from the four categories (asthenopic, ocular surface-related, visual, and extra-ocular) to the intermediate variable were 0.842 ± 0.024, 0.657 ± 0.018, 0.787 ± 0.027, and 0.823 ± 0.022, respectively, and “asthenopic” and “extra-ocular” were found to be relatively strong symptoms for CVS. Thus, we believe that SEM has the potential to enhance our understanding of complex phenotypes, such as syndromes, by genetically unraveling the relationship between observed symptoms. SEM is expected to be a core technique for performing a series of association analyses from GWAS to PheWAS.

## Supplementary Information

Supplementary Figures.

## Data Availability

The datasets used and/or analyzed during the current study are available from the corresponding authors on reasonable request.
