# Supplementary material for: A web-based survey on various symptoms of computer vision syndrome and the genetic understanding based on a multi-trait genome-wide association study
Source: Sci Rep. 2021 May 3;11:9446. doi: 10.1038/s41598-021-88827-y (PMC8093242; doi:10.1038/s41598-021-88827-y)
Supplement: Supplementary file 1 — Supplementary Figures. [file 41598_2021_88827_MOESM1_ESM.doc]

**A web-based survey on various symptoms of computer vision syndrome and the genetic understanding based on a multi-trait genome-wide association study**

Keito Yoshimura^1,*^, Yuji Morita^2,*^, Kenji Konomi^3^, Sachiko Ishida^1^, Daisuke Fujiwara^4^, Keisuke Kobayashi^1,*^, and Masami Tanaka^1^

^1^DeNA Life Science, Inc., Tokyo, Japan.

^2^Kirin Central Research Institute, Kirin Holdings Company, Limited, Yokohama, Japan.

^3^Clinical and Translational Research Center, Keio University Hospital, Tokyo, Japan.

^4^Health Science Department, Kirin Holdings Company, Limited, Tokyo, Japan.

* Correspondence and requests for materials should be address to Keito Yoshimura, Yuji Morita, and Keisuke Kobayashi.

**Supplementary Figures**

Supplementary Figure S1.

| All population | Only East Asian population |
| --- | --- |
| 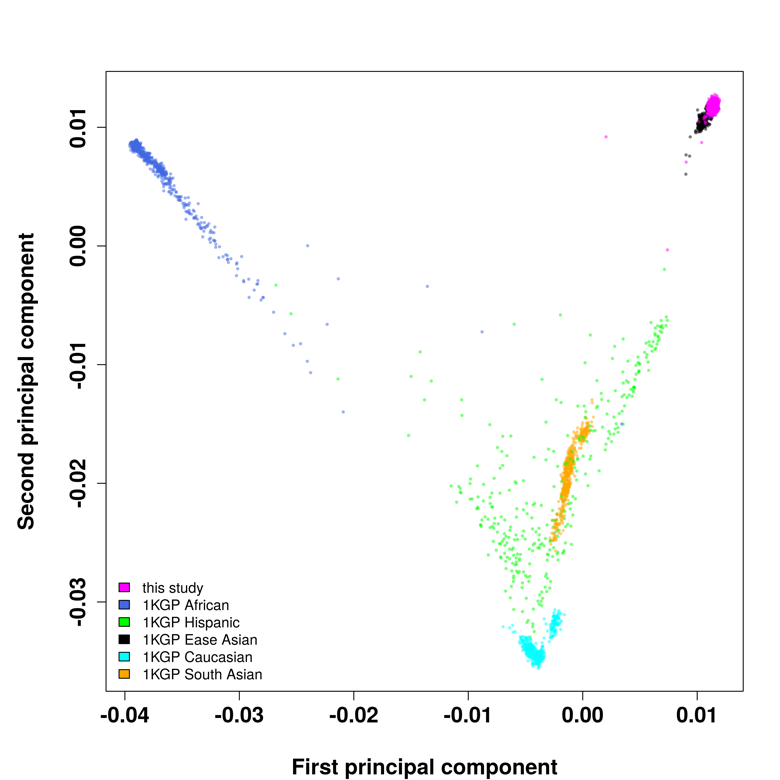 | 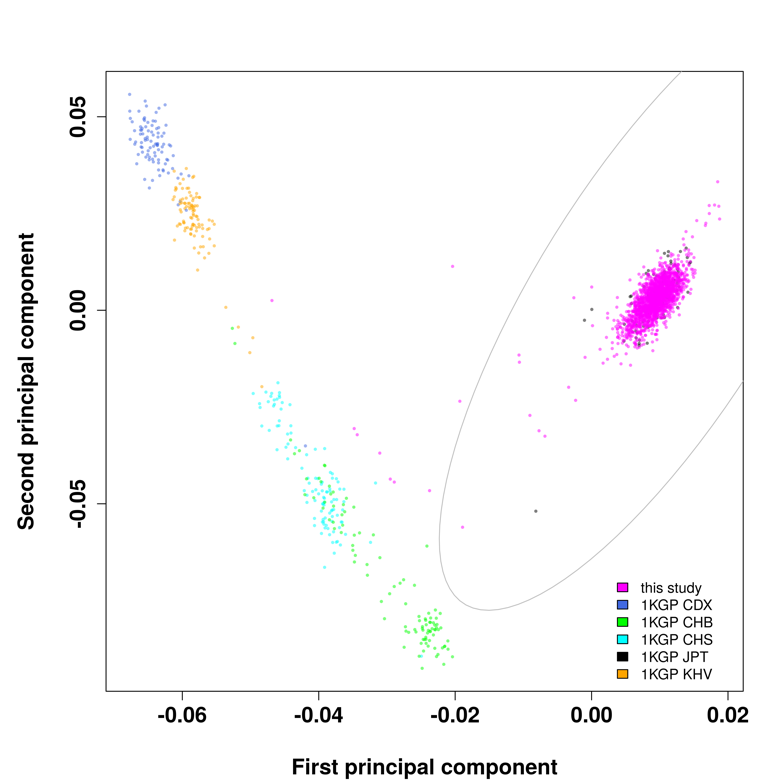 |

PCA plots of population structure analysis. PCA using PLINK was conducted after filtering SNPs with the following command options: *plink –indep-pairwise 50 5 0.5 –maf 0.1*. It was confirmed that the positional relationships within the 1KGP population on the subspace was consistent with that of previous studies.^1^ The grey ellipse in only East Asian population shows a range of 10 × standard deviation of 1KGP JPT population, and study samples outside of the ellipse were removed in the following analysis.

Supplementary Figure S2.


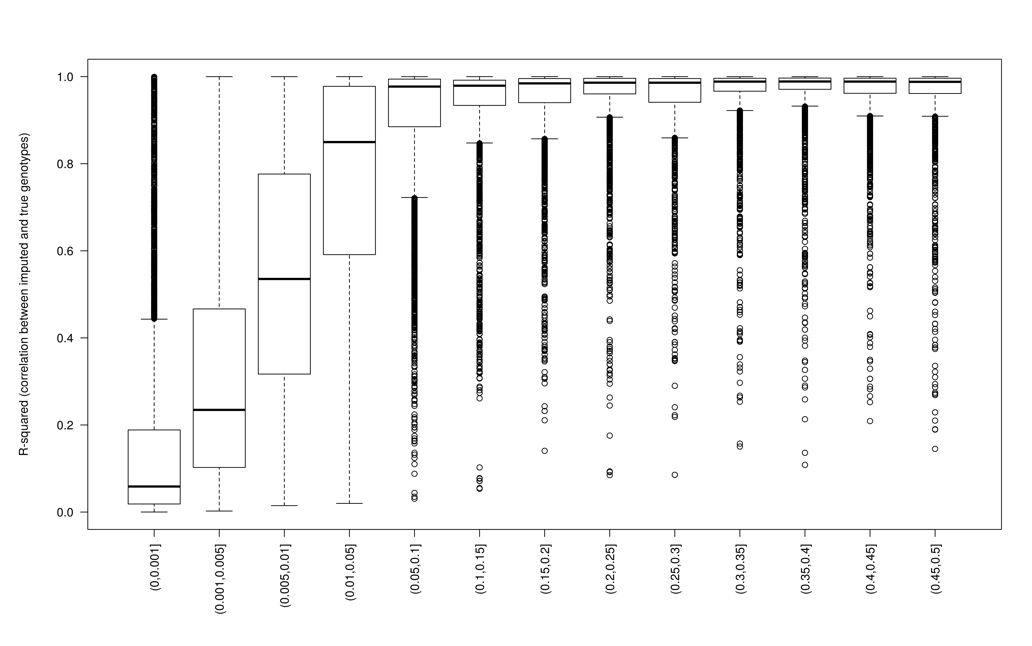


Box plot of R-squared by a range of MAF for assessment of imputation performance. The boxes range from the first to the third quartile, the central line is the median, and the whiskers range from minimum to maximum values.

Supplementary Figure S3.


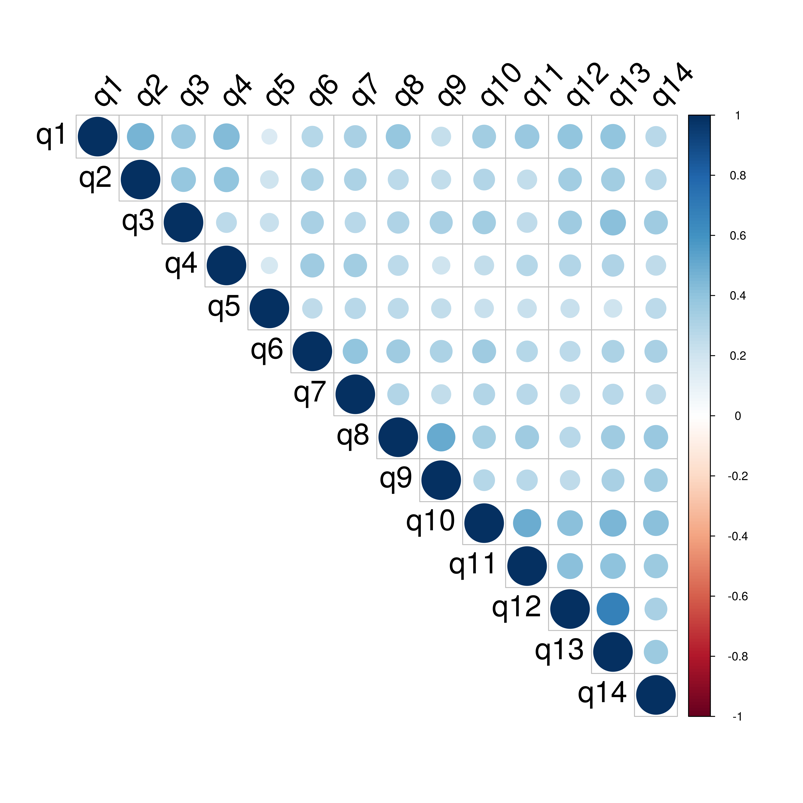


Heatmap of correlation matrix between questionnaire items.

Supplementary Figure S4.


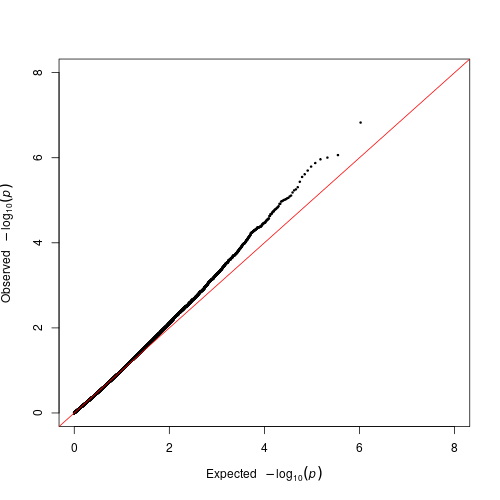


Q-Q plot of results of multi-trait GWAS.

Supplementary Figure S5.

| Chr1: 47750000-48750000 | Chr1: 94500000-95500000 |
| --- | --- |
|  |  |
| Chr2: 1000000-2000000 | Chr3: 118000000-119000000 |
|  |  |
| Chr7: 3000000-4000000 | Chr7: 77750000-78750000 |
|  |  |

| Chr10: 53500000-54500000 | Chr11: 21250000-22250000 |
| --- | --- |
|  |  |
| Chr11: 109500000-110500000 | Chr17: 8000000-9000000 |
|  |  |
| Chr17: 76750000-77750000 | Chr18: 36000000-37000000 |
|  |  |

Regional plots of identified loci via multi-trait GWAS. The -log_10_(p-value) of each SNPs is indicated by a dot. The purple diamond-shaped dots represent the most significantly associated with the trait in each region. The color intensity indicated the extent of linkage disequilibrium index (R-squared) with the most significant SNP (purple dot). Estimated recombination rates from the 1KGP Asian population (Nov 2014) were shown as light-blue lines.

Supplementary Figure S6.

| “Asthenopic” category (by gwsem) | “Asthenopic” category (by PLINK) |
| --- | --- |
| 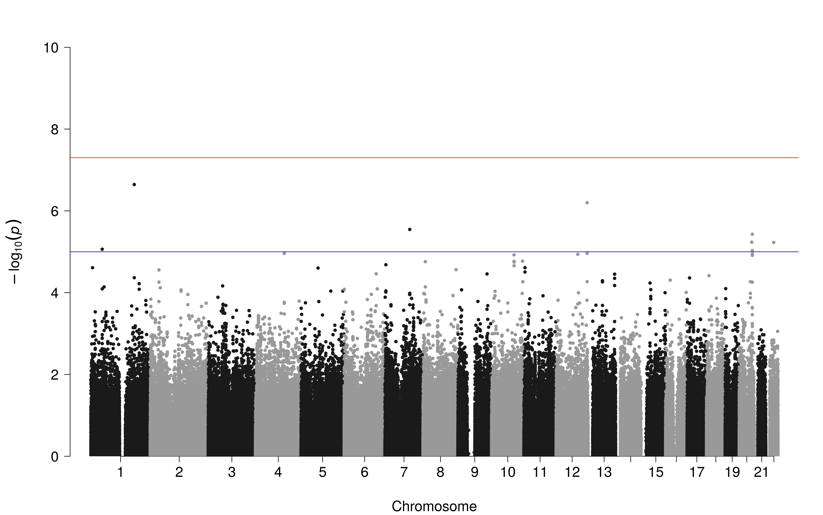 | 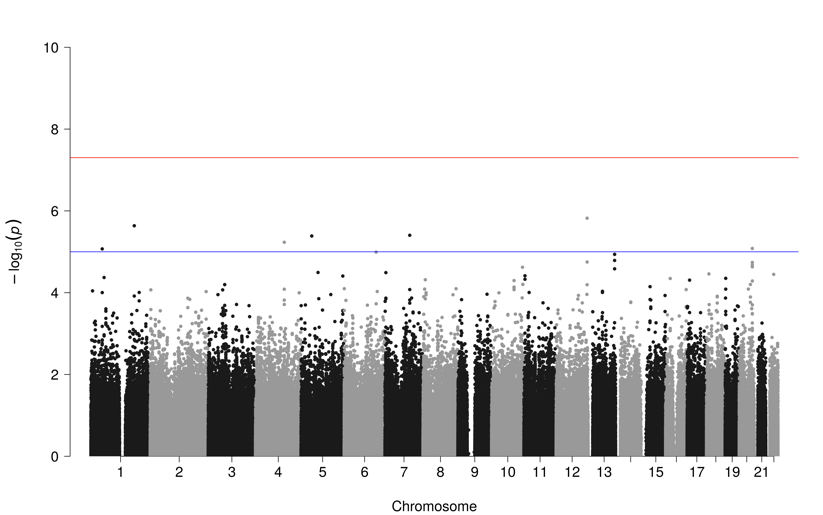 |
| “Ocular surface-related” category (by gwsem) | “Ocular surface-related” category (by PLINK) |
| 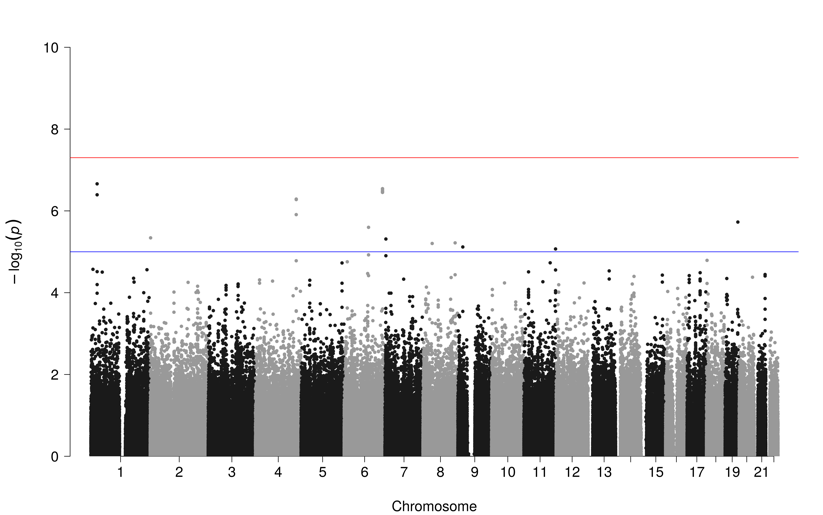 | 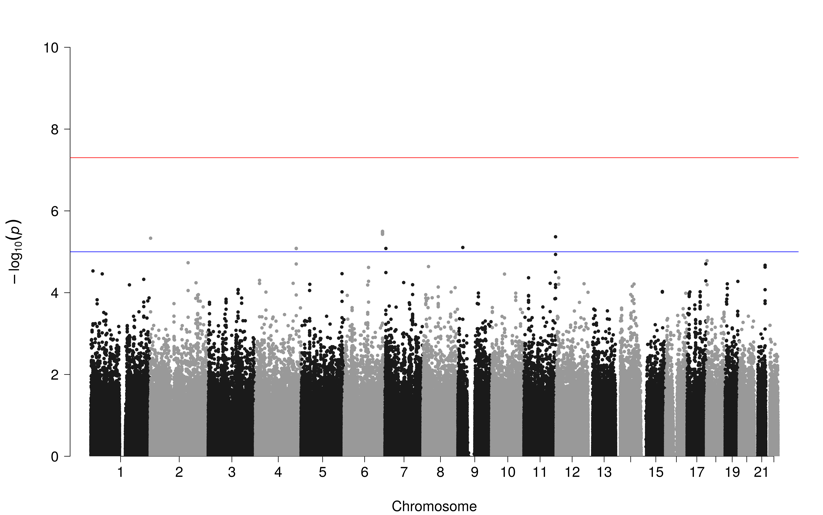 |
| “Visual” category (by gwsem) | “Visual” category (by PLINK) |
| 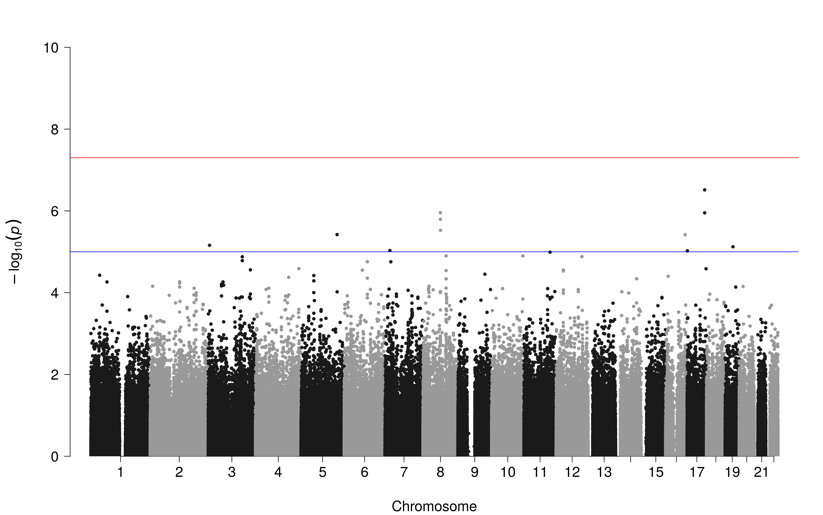 | 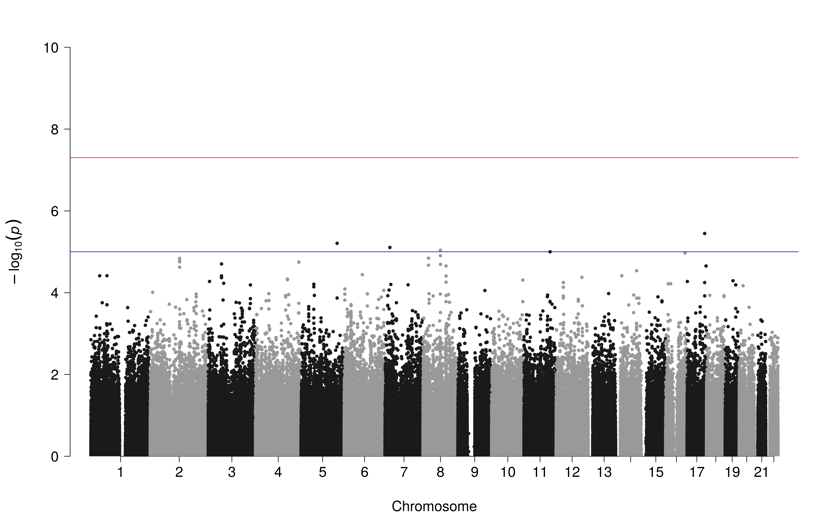 |

| “Extra–ocular” category (by gwsem) | “Extra–ocular” category (by PLINK) |
| --- | --- |
| 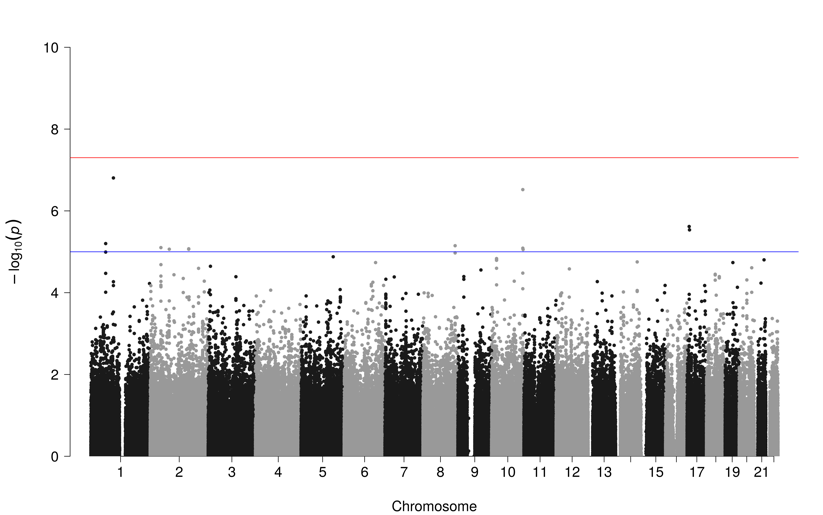 | 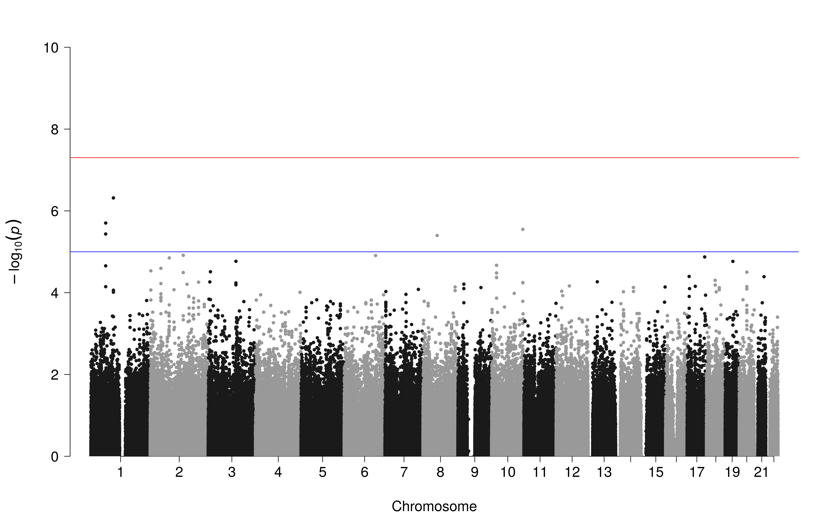 |

Manhattan plots of results of single-trait GWAS.

**Supplementary References**

1 Zhou, *X. et a*l. Identification of genetic risk factors in the Chinese population implicates a role of immune system in Alzheimer's disease pathogenesis*. Proc Natl Acad Sci U S* **A 1**15, 1697-1706, doi:10.1073/pnas.1715554115 (2018).
